# Crystal structure of 1-benzyl-3-methyl-1*H*-imidazolium hexa­fluorido­phosphate

**DOI:** 10.1107/S1600536814024301

**Published:** 2014-11-12

**Authors:** Patrick C. Hillesheim, Kent A. Scipione

**Affiliations:** a Mississippi State University, Department of Chemistry, 1115 Hand Lab, Box 9573, Mississippi State, MS 39762, USA

**Keywords:** crystal structure, imidazolium, hexa­fluorido­phosphate, hydrogen bonding, π–π inter­actions

## Abstract

In the title salt, C_11_H_13_N_2_^+^·PF_6_^−^, the dihedral angle between the planes of the imidazole and benzene rings is 84.72 (4)°. In the crystal, C—H⋯F inter­actions connect the cation and anion pairs into a three-dimensional network. Weak π–π inter­actions are observed between the imidazolium ring and the aromatic benzene ring of an adjacent mol­ecule with C⋯C and C⋯N distances ranging from 3.3714 (16) to 3.4389 (15) Å.

## Related literature   

For related structures containing imidazolium rings bearing *N*-benzyl groups, see: Haque *et al.* (2012 ▶); Jiang (2009 ▶); Lu *et al.* (2010 ▶); Pi *et al.* (2009 ▶). For an overview of applications for ionic liquids, see: Plechkova & Seddon (2008 ▶). For applications of benzyl-containing ionic liquids, see: Mahurin *et al.* (2011 ▶). For the synthesis of the title compound, see: Shkrob *et al.* (2013 ▶). For use of imidazolium compounds as carbene precursors, see: Scholl *et al.* (1999 ▶).
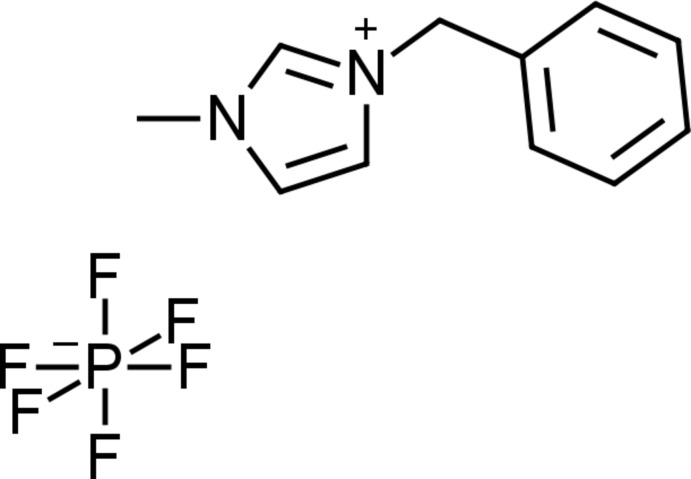


## Experimental   

### Crystal data  

C_11_H_13_N_2_^+^·F_6_P^−^
*M* *_r_* = 318.20
Monoclinic, 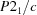

*a* = 10.4989 (3) Å
*b* = 11.2755 (3) Å
*c* = 11.9769 (3) Å
β = 109.926 (1)°
*V* = 1332.95 (6) Å^3^
*Z* = 4
Mo *K*α radiation
μ = 0.27 mm^−1^
*T* = 100 K
0.45 × 0.27 × 0.12 mm

### Data collection  

Bruker APEXII CCD diffractometer
Absorption correction: multi-scan (*SADABS*; Bruker, 2014 ▶) *T*_min_ = 0.906, *T*_max_ = 0.991
42589 measured reflections
3188 independent reflections
2746 reflections with *I* > 2σ(*I*)
*R*_int_ = 0.055

### Refinement  

*R*[*F* ^2^ > 2σ(*F* ^2^)] = 0.031
*wR*(*F* ^2^) = 0.083
*S* = 1.07
3188 reflections
182 parameters
H-atom parameters constrained
Δρ_max_ = 0.27 e Å^−3^
Δρ_min_ = −0.42 e Å^−3^

### 

Data collection: *APEX2* (Bruker, 2014 ▶); cell refinement: *SAINT* (Bruker, 2013 ▶); data reduction: *SAINT*; program(s) used to solve structure: *SHELXT* (Sheldrick, 2008 ▶); program(s) used to refine structure: *SHELXL* (Sheldrick, 2008 ▶); molecular graphics: *OLEX2* (Dolomanov *et al.*, 2009 ▶); software used to prepare material for publication: *OLEX2*.

## Supplementary Material

Crystal structure: contains datablock(s) I. DOI: 10.1107/S1600536814024301/zl2605sup1.cif

Structure factors: contains datablock(s) I. DOI: 10.1107/S1600536814024301/zl2605Isup2.hkl

Supporting information file. DOI: 10.1107/S1600536814024301/zl2605Isup3.mol

**Fig. 1.**
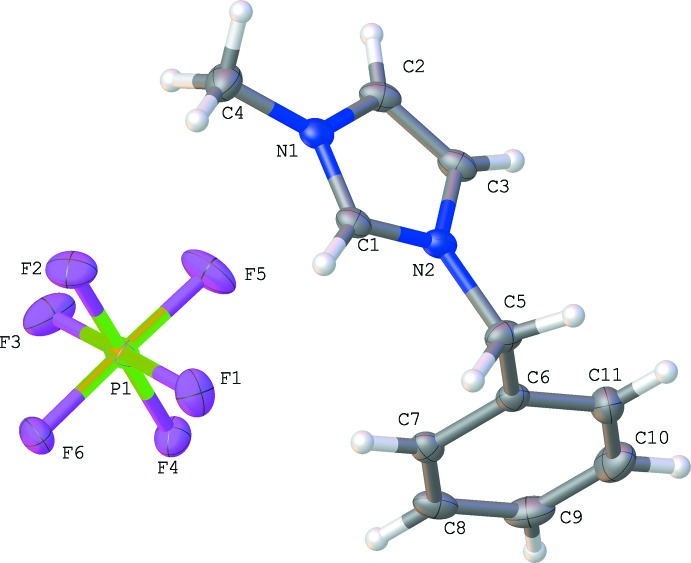
The molecular structure of the title compound with 50% probability ellipsoids. Nitrogen atoms shown in blue, carbon in grey, fluorine in pink, and phosphorous in green.

. DOI: 10.1107/S1600536814024301/zl2605fig1.tif
The mol­ecular structure of the title compound with 50% probability ellipsoids. Nitro­gen atoms shown in blue, carbon in grey, fluorine in pink, and phospho­rous in green.

**Fig. 2.**
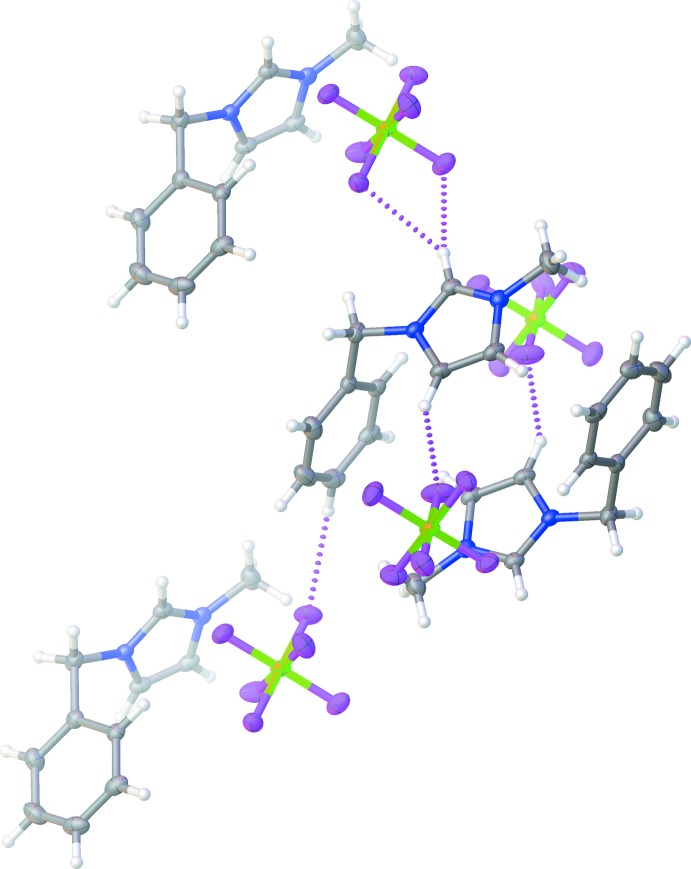
Diagram of the hydrogen bonding observed in the title compound shown as pink dotted lines.

. DOI: 10.1107/S1600536814024301/zl2605fig2.tif
Diagram of the hydrogen bonding observed in the title compound shown as pink dotted lines.

CCDC reference: 1032692

Additional supporting information:  crystallographic information; 3D view; checkCIF report

## Figures and Tables

**Table 1:** Hydrogen-bond geometry (, )

*D*H*A*	*D*H	H*A*	*D* *A*	*D*H*A*
C1H1F3^i^	0.95	2.23	3.1503(14)	164
C1H1F4^i^	0.95	2.61	3.2860(14)	129
C3H3F5^ii^	0.95	2.30	3.1456(15)	148
C9H9F2^iii^	0.95	2.60	3.2680(16)	128

Symmetry codes: (i) 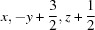; (ii) 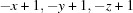; (iii) 
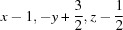
.

## supplementary crystallographic information

### S1. Structural commentary

Ionic liquids are a class of materials which have found a wide range of applications in recent years (Plechkova & Seddon, 2008). In addition to their applications involving ionic liquids, imidazolium based salts are also commonly employed as starting materials for carbene ligands, perhaps most famously for their use in the so called Grubbs catalyst (Scholl *et al.*, 1999). For both ionic liquids and carbene ligands, cationic nitro­gen containing heterocycles are the dominant structural motif, providing an ideal combination of chemical and physical properties useful in both instances. Electronic and structural factors play a large role in the fine tuning of both ionic liquids and carbenes, thus the structure reported herein will provide a useful analysis of this common, yet unreported structure.

The asymmetric unit of the title compound contains one cation-anion pair (Fig. 1). The dihedral angle of the benzene ring and the imidazolium ring is 84.72° (4), see table 1. Crystal packing appears to be stabilized by the presence of several C—H···F inter­actions summarized in table 2 and shown in Figure 2. There are several close contacts between C7 and C8 of the benzene ring and the symmetry generated imidazole ring (symmetry operator 1-x,1/2+y,1.5-z) with C···C and C···N distances ranging from 3.3714 (16) to 3.4389 (15) (for C7···C2 and C8···N1 respectively). These inter­actions likely point towards the presence of a weak, highly slipped and tilted π-π inter­action. No classical centroid-centroid π stacking was observed in the lattice.

### S2. Synthesis and crystallization

The title compound was synthesized according to established literature procedures (Shkrob *et al.*, 2013). Single crystals suitable for diffraction were grown by slow solvent evaporation from an ethano­lic solution of the title compound.

### S3. Refinement

Crystal data, data collection and structure refinement details are summarized in Table 1. H atoms were included at calculated positions using a riding model, with aromatic, methyl­ene, and methyl C—H bond lengths of 0.95, 0.99 and 0.98Å, respectively. The U_iso_(H) values were fixed at 1.5U_eq_(C) for methyl H atoms, and 1.2U_eq_(C) for all other C atoms.

### Crystal data

**Table:**

C_11_H_13_N_2_^+^·F_6_P^−^	*F*(000) = 648
*M**_r_* = 318.20	*D*_x_ = 1.586 Mg m^−^^3^
Monoclinic, *P*2_1_/*c*	Mo *K*α radiation, λ = 0.71073 Å
*a* = 10.4989 (3) Å	Cell parameters from 9735 reflections
*b* = 11.2755 (3) Å	θ = 2.6–27.9°
*c* = 11.9769 (3) Å	µ = 0.27 mm^−^^1^
β = 109.926 (1)°	*T* = 100 K
*V* = 1332.95 (6) Å^3^	Plates, colourless
*Z* = 4	0.45 × 0.27 × 0.12 mm

### Data collection

**Table:**

Bruker APEXII CCD diffractometer	2746 reflections with *I* > 2σ(*I*)
Detector resolution: 8.33 pixels mm^-1^	*R*_int_ = 0.055
combination of ω and φ–scans	θ_max_ = 27.9°, θ_min_ = 2.1°
Absorption correction: multi-scan (*SADABS*; Bruker, 2014)	*h* = −13→13
*T*_min_ = 0.906, *T*_max_ = 0.991	*k* = −14→14
42589 measured reflections	*l* = −15→15
3188 independent reflections	

### Refinement

**Table:**

Refinement on *F*^2^	Primary atom site location: structure-invariant direct methods
Least-squares matrix: full	Hydrogen site location: inferred from neighbouring sites
*R*[*F*^2^ > 2σ(*F*^2^)] = 0.031	H-atom parameters constrained
*wR*(*F*^2^) = 0.083	*w* = 1/[σ^2^(*F*_o_^2^) + (0.0416*P*)^2^ + 0.409*P*] where *P* = (*F*_o_^2^ + 2*F*_c_^2^)/3
*S* = 1.07	(Δ/σ)_max_ = 0.001
3188 reflections	Δρ_max_ = 0.27 e Å^−^^3^
182 parameters	Δρ_min_ = −0.42 e Å^−^^3^
0 restraints	

### Special details

**Table:**

Geometry. All e.s.d.'s (except the e.s.d. in the dihedral angle between two l.s. planes) are estimated using the full covariance matrix. The cell e.s.d.'s are taken into account individually in the estimation of e.s.d.'s in distances, angles and torsion angles; correlations between e.s.d.'s in cell parameters are only used when they are defined by crystal symmetry. An approximate (isotropic) treatment of cell e.s.d.'s is used for estimating e.s.d.'s involving l.s. planes.

### Fractional atomic coordinates and isotropic or equivalent isotropic displacement parameters (Å^2^)

**Table:**

	*x*	*y*	*z*	*U*_iso_*/*U*_eq_	
N1	0.71519 (10)	0.47646 (9)	0.73967 (9)	0.0195 (2)	
N2	0.51532 (10)	0.53231 (9)	0.72989 (9)	0.0177 (2)	
C1	0.64714 (12)	0.54719 (10)	0.78756 (10)	0.0188 (2)	
H1	0.6860	0.5997	0.8523	0.023*	
C2	0.62396 (13)	0.41476 (11)	0.64770 (11)	0.0219 (3)	
H2	0.6453	0.3583	0.5979	0.026*	
C3	0.49888 (13)	0.44926 (10)	0.64138 (11)	0.0210 (2)	
H3	0.4154	0.4217	0.5866	0.025*	
C4	0.86303 (13)	0.46510 (13)	0.77897 (13)	0.0296 (3)	
H4A	0.8957	0.4942	0.7165	0.044*	
H4B	0.8883	0.3816	0.7954	0.044*	
H4C	0.9039	0.5119	0.8513	0.044*	
C5	0.40534 (13)	0.59543 (11)	0.75594 (11)	0.0226 (3)	
H5A	0.4451	0.6509	0.8229	0.027*	
H5B	0.3491	0.5373	0.7803	0.027*	
C6	0.31700 (12)	0.66383 (11)	0.64927 (11)	0.0190 (2)	
C7	0.36887 (12)	0.76192 (10)	0.60829 (10)	0.0196 (2)	
H7	0.4604	0.7850	0.6469	0.023*	
C8	0.28734 (14)	0.82614 (11)	0.51126 (11)	0.0244 (3)	
H8	0.3230	0.8933	0.4841	0.029*	
C9	0.15414 (15)	0.79246 (13)	0.45415 (12)	0.0303 (3)	
H9	0.0986	0.8364	0.3877	0.036*	
C10	0.10175 (14)	0.69493 (14)	0.49367 (14)	0.0334 (3)	
H10	0.0105	0.6716	0.4540	0.040*	
C11	0.18284 (13)	0.63074 (12)	0.59182 (13)	0.0276 (3)	
H11	0.1464	0.5644	0.6194	0.033*	
P1	0.76866 (3)	0.81119 (3)	0.62758 (3)	0.01967 (10)	
F1	0.71267 (8)	0.80793 (7)	0.73665 (7)	0.0306 (2)	
F2	0.90222 (8)	0.74247 (7)	0.70492 (7)	0.0333 (2)	
F3	0.82307 (9)	0.81640 (8)	0.51809 (7)	0.0363 (2)	
F4	0.63354 (7)	0.88092 (7)	0.54915 (7)	0.02663 (18)	
F5	0.69426 (10)	0.68792 (7)	0.58054 (8)	0.0392 (2)	
F6	0.84023 (8)	0.93566 (7)	0.67485 (8)	0.0310 (2)	

### Atomic displacement parameters (Å^2^)

**Table:**

	*U*^11^	*U*^22^	*U*^33^	*U*^12^	*U*^13^	*U*^23^
N1	0.0232 (5)	0.0161 (5)	0.0195 (5)	0.0017 (4)	0.0076 (4)	0.0036 (4)
N2	0.0232 (5)	0.0154 (4)	0.0148 (5)	0.0016 (4)	0.0069 (4)	0.0008 (4)
C1	0.0253 (6)	0.0146 (5)	0.0157 (5)	−0.0003 (4)	0.0061 (5)	0.0018 (4)
C2	0.0336 (7)	0.0163 (5)	0.0161 (6)	0.0028 (5)	0.0089 (5)	0.0001 (4)
C3	0.0293 (6)	0.0163 (5)	0.0155 (6)	−0.0010 (5)	0.0051 (5)	−0.0014 (4)
C4	0.0227 (6)	0.0292 (7)	0.0372 (8)	0.0041 (5)	0.0106 (6)	0.0070 (6)
C5	0.0271 (6)	0.0219 (6)	0.0224 (6)	0.0049 (5)	0.0132 (5)	0.0034 (5)
C6	0.0215 (6)	0.0171 (5)	0.0203 (6)	0.0023 (4)	0.0095 (5)	−0.0015 (4)
C7	0.0239 (6)	0.0183 (5)	0.0178 (6)	−0.0010 (5)	0.0088 (5)	−0.0033 (5)
C8	0.0387 (7)	0.0165 (6)	0.0200 (6)	0.0050 (5)	0.0127 (6)	−0.0005 (5)
C9	0.0345 (7)	0.0288 (7)	0.0235 (7)	0.0145 (6)	0.0043 (6)	−0.0007 (5)
C10	0.0206 (6)	0.0375 (8)	0.0368 (8)	0.0035 (5)	0.0030 (6)	−0.0075 (6)
C11	0.0236 (6)	0.0239 (6)	0.0364 (8)	−0.0024 (5)	0.0115 (6)	−0.0017 (6)
P1	0.02424 (17)	0.01596 (16)	0.01680 (17)	−0.00009 (11)	0.00438 (13)	0.00205 (11)
F1	0.0351 (4)	0.0369 (5)	0.0204 (4)	−0.0067 (3)	0.0103 (3)	0.0022 (3)
F2	0.0365 (5)	0.0288 (4)	0.0271 (4)	0.0110 (3)	0.0013 (3)	0.0054 (3)
F3	0.0406 (5)	0.0465 (5)	0.0264 (4)	0.0185 (4)	0.0175 (4)	0.0112 (4)
F4	0.0224 (4)	0.0296 (4)	0.0265 (4)	0.0025 (3)	0.0065 (3)	0.0077 (3)
F5	0.0572 (6)	0.0205 (4)	0.0282 (5)	−0.0093 (4)	−0.0007 (4)	−0.0038 (3)
F6	0.0262 (4)	0.0201 (4)	0.0450 (5)	−0.0052 (3)	0.0099 (4)	−0.0014 (3)

### Geometric parameters (Å, º)

**Table:**

N1—C1	1.3247 (16)	C6—C11	1.3914 (18)
N1—C2	1.3768 (16)	C7—H7	0.9500
N1—C4	1.4660 (16)	C7—C8	1.3891 (17)
N2—C1	1.3301 (16)	C8—H8	0.9500
N2—C3	1.3805 (15)	C8—C9	1.384 (2)
N2—C5	1.4774 (15)	C9—H9	0.9500
C1—H1	0.9500	C9—C10	1.383 (2)
C2—H2	0.9500	C10—H10	0.9500
C2—C3	1.3468 (18)	C10—C11	1.396 (2)
C3—H3	0.9500	C11—H11	0.9500
C4—H4A	0.9800	P1—F1	1.6053 (8)
C4—H4B	0.9800	P1—F2	1.5935 (8)
C4—H4C	0.9800	P1—F3	1.6000 (8)
C5—H5A	0.9900	P1—F4	1.6139 (8)
C5—H5B	0.9900	P1—F5	1.5999 (8)
C5—C6	1.5098 (17)	P1—F6	1.6016 (8)
C6—C7	1.3939 (17)		
			
C1—N1—C2	108.65 (10)	C6—C7—H7	119.8
C1—N1—C4	125.58 (11)	C8—C7—C6	120.33 (12)
C2—N1—C4	125.77 (11)	C8—C7—H7	119.8
C1—N2—C3	108.67 (10)	C7—C8—H8	120.0
C1—N2—C5	125.40 (10)	C9—C8—C7	120.08 (12)
C3—N2—C5	125.93 (11)	C9—C8—H8	120.0
N1—C1—N2	108.57 (10)	C8—C9—H9	119.9
N1—C1—H1	125.7	C10—C9—C8	120.11 (13)
N2—C1—H1	125.7	C10—C9—H9	119.9
N1—C2—H2	126.3	C9—C10—H10	120.0
C3—C2—N1	107.35 (11)	C9—C10—C11	120.05 (13)
C3—C2—H2	126.3	C11—C10—H10	120.0
N2—C3—H3	126.6	C6—C11—C10	120.10 (13)
C2—C3—N2	106.76 (11)	C6—C11—H11	119.9
C2—C3—H3	126.6	C10—C11—H11	119.9
N1—C4—H4A	109.5	F1—P1—F4	89.53 (4)
N1—C4—H4B	109.5	F2—P1—F1	90.55 (5)
N1—C4—H4C	109.5	F2—P1—F3	90.29 (5)
H4A—C4—H4B	109.5	F2—P1—F4	179.90 (5)
H4A—C4—H4C	109.5	F2—P1—F5	90.55 (5)
H4B—C4—H4C	109.5	F2—P1—F6	90.39 (4)
N2—C5—H5A	109.3	F3—P1—F1	179.05 (5)
N2—C5—H5B	109.3	F3—P1—F4	89.63 (4)
N2—C5—C6	111.49 (10)	F3—P1—F6	90.11 (5)
H5A—C5—H5B	108.0	F5—P1—F1	89.80 (5)
C6—C5—H5A	109.3	F5—P1—F3	90.64 (5)
C6—C5—H5B	109.3	F5—P1—F4	89.51 (5)
C7—C6—C5	120.15 (11)	F5—P1—F6	178.80 (5)
C11—C6—C5	120.52 (11)	F6—P1—F1	89.44 (4)
C11—C6—C7	119.32 (12)	F6—P1—F4	89.56 (4)
			
N1—C2—C3—N2	0.15 (13)	C5—N2—C1—N1	−179.75 (10)
N2—C5—C6—C7	−67.85 (14)	C5—N2—C3—C2	179.52 (11)
N2—C5—C6—C11	112.96 (13)	C5—C6—C7—C8	−179.04 (11)
C1—N1—C2—C3	−0.35 (13)	C5—C6—C11—C10	179.65 (12)
C1—N2—C3—C2	0.11 (13)	C6—C7—C8—C9	−0.45 (18)
C1—N2—C5—C6	121.23 (12)	C7—C6—C11—C10	0.45 (19)
C2—N1—C1—N2	0.42 (13)	C7—C8—C9—C10	0.14 (19)
C3—N2—C1—N1	−0.33 (13)	C8—C9—C10—C11	0.5 (2)
C3—N2—C5—C6	−58.10 (15)	C9—C10—C11—C6	−0.8 (2)
C4—N1—C1—N2	−179.05 (11)	C11—C6—C7—C8	0.16 (18)
C4—N1—C2—C3	179.12 (11)		

### Hydrogen-bond geometry (Å, º)

**Table:**

*D*—H···*A*	*D*—H	H···*A*	*D*···*A*	*D*—H···*A*
C1—H1···F3^i^	0.95	2.23	3.1503 (14)	164
C1—H1···F4^i^	0.95	2.61	3.2860 (14)	129
C3—H3···F5^ii^	0.95	2.30	3.1456 (15)	148
C9—H9···F2^iii^	0.95	2.60	3.2680 (16)	128

Symmetry codes: (i) *x*, −*y*+3/2, *z*+1/2; (ii) −*x*+1, −*y*+1, −*z*+1; (iii) *x*−1, −*y*+3/2, *z*−1/2.
