# Supplementary figures and images for: Crystal structure of 1-benzyl-3-methyl-1H-imidazolium hexa­fluorido­phosphate
Source: Acta Crystallogr Sect E Struct Rep Online. 2014 Nov 12;70(Pt 12):o1248–9. doi: 10.1107/S1600536814024301 (PMC4257422; doi:10.1107/S1600536814024301)

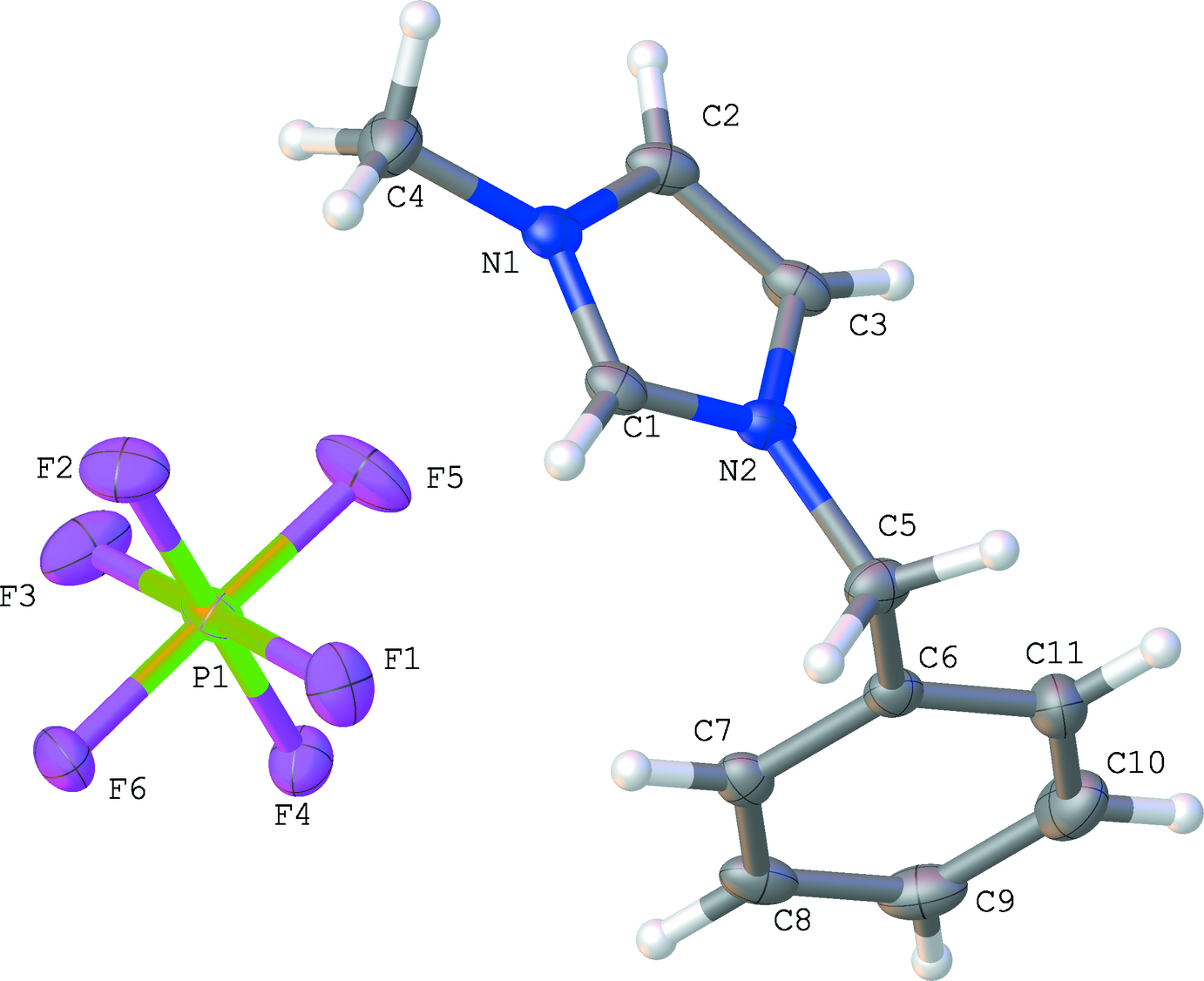

Supplement: Supplementary file 4 [file e-70-o1248-fig1.tif]

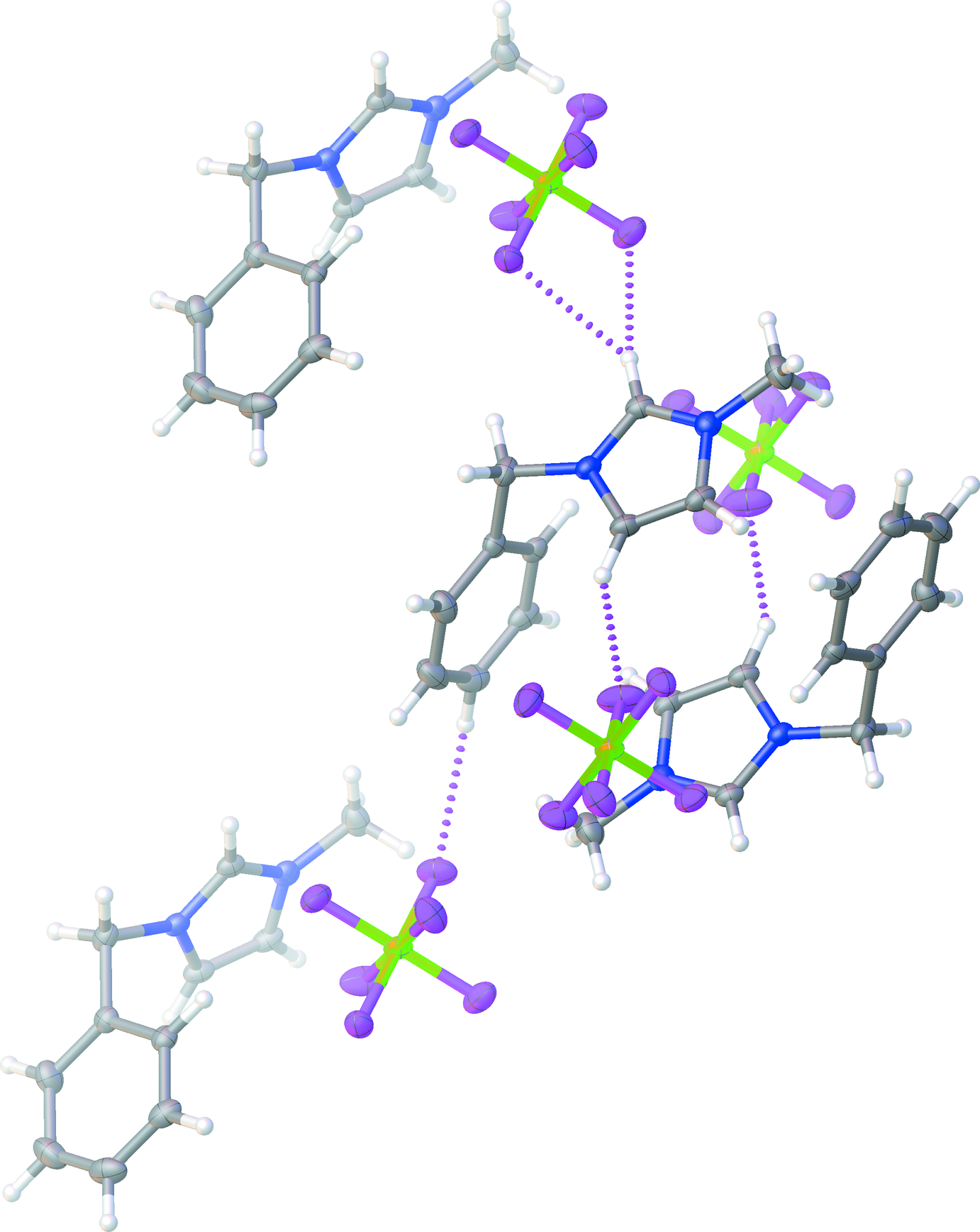

Supplement: Supplementary file 5 [file e-70-o1248-fig2.tif]
